# Improvements in Bladder Function Following Activity-Based Recovery Training With Epidural Stimulation After Chronic Spinal Cord Injury

**DOI:** 10.3389/fnsys.2020.614691

**Published:** 2021-01-05

**Authors:** April N. Herrity, Sevda C. Aslan, Beatrice Ugiliweneza, Ahmad Z. Mohamed, Charles H. Hubscher, Susan J. Harkema

**Affiliations:** ^1^Kentucky Spinal Cord Injury Research Center, University of Louisville, Louisville, KY, United States; ^2^Department of Neurological Surgery, University of Louisville, Louisville, KY, United States; ^3^Department of Urology, University of Louisville, Louisville, KY, United States; ^4^Department of Anatomical Sciences and Neurobiology, University of Louisville, Louisville, KY, United States

**Keywords:** lower urinary tract, urodynamics, neurogenic bladder, neuromodulation, locomotor training, cardiovascular

## Abstract

Spinal cord injury (SCI) results in profound neurologic impairment with widespread deficits in sensorimotor and autonomic systems. Voluntary and autonomic control of bladder function is disrupted resulting in possible detrusor overactivity, low compliance, and uncoordinated bladder and external urethral sphincter contractions impairing storage and/or voiding. Conservative treatments managing neurogenic bladder post-injury, such as oral pharmacotherapy and catheterization, are important components of urological surveillance and clinical care. However, as urinary complications continue to impact long-term morbidity in this population, additional therapeutic and rehabilitative approaches are needed that aim to improve function by targeting the recovery of underlying impairments. Several human and animal studies, including our previously published reports, have documented gains in bladder function due to activity-based recovery strategies, such as locomotor training. Furthermore, epidural stimulation of the spinal cord (scES) combined with intense activity-based recovery training has been shown to produce volitional lower extremity movement, standing, as well as improve the regulation of cardiovascular function. In our center, several participants anecdotally reported improvements in bladder function as a result of training with epidural stimulation configured for motor systems. Thus, in this study, the effects of activity-based recovery training in combination with scES were tested on bladder function, resulting in improvements in overall bladder storage parameters relative to a control cohort (no intervention). However, elevated blood pressure elicited during bladder distention, characteristic of autonomic dysreflexia, was not attenuated with training. We then examined, in a separate, large cross-sectional cohort, the interaction between detrusor pressure and blood pressure at maximum capacity, and found that the functional relationship between urinary bladder distention and blood pressure regulation is disrupted. Regardless of one’s bladder emptying method (indwelling suprapubic catheter vs. intermittent catheterization), autonomic instability can play a critical role in the ability to improve bladder storage, with SCI enhancing the vesico-vascular reflex. These results support the role of intersystem stimulation, integrating scES for both bladder and cardiovascular function to further improve bladder storage.

## Introduction

Over 1.4 million Americans have a spinal cord injury (SCI; Armour et al., 2016), with 70–84% having at least some degree of bladder dysfunction (Hamid et al., 2018). Injury above the sacral cord results in a loss of volitional control of micturition consistent with an upper motor neuron-type injury. The resulting neurogenic bladder is characterized by detrusor overactivity and detrusor-sphincter dyssynergia, where simultaneous detrusor and urinary sphincter contractions lead to high bladder pressure and insufficient emptying (de Groat and Yoshimura, 2010). Functional impairments of the lower urinary tract (LUT) is an area of highest priority, as it has a dramatic negative impact on overall health and quality of life (Anderson, 2004; Ditunno et al., 2008; Piatt et al., 2016). Major urological concerns contributing to increased morbidity and mortality include repeated LUT infections that can lead to sepsis, chronic vesicoureteral reflux and hydronephrosis with progression to renal failure as a result of high-intravesical pressures, and inter-related cardiovascular complications such as autonomic dysreflexia (Van Kerrebroeck et al., 1993; Zeilig et al., 2000; Hagen et al., 2011) that limits bladder storage (Hubscher et al., 2018). Standard management of LUT dysfunction post-SCI includes a combination of pharmacological approaches to reduce bladder over-activity and pressure and catheter-based management to empty the bladder. While these approaches can decrease urinary complications in those who can tolerate medications as well as perform urethral catheterizations, many individuals performing intermittent catheterization do not remain on this method long-term, with some having surgical urinary diversion, either continent or to a stoma device, and most transitioning to an indwelling catheter, a management strategy associated with a high degree of medical complications and hospitalizations (Cameron et al., 2010, 2011). There is a critical need for a successful intervention that aims to restore function, as even though the standard of care manages the many limitations attributed to secondary complications after injury, it does not access the inherent ability of the nervous system to recover function. Current bladder management approaches commonly require life-long maintenance, and have adverse side effects leading to recurring illness and reduced quality of life (Benevento and Sipski, 2002).

Activity-based recovery therapy, such as locomotor training, which engages lumbosacral spinal networks below the level of injury to retrain the nervous system to recover a specific motor task, is an effective rehabilitation strategy for improving post-SCI motor outcomes (Dietz and Harkema, 2004; Behrman et al., 2005; Harkema et al., 2012; Jones et al., 2014; Kaiser et al., 2020), as well as improving autonomic responses (Harkema et al., 2008; Terson de Paleville et al., 2013; Onushko et al., 2019), including bladder, bowel, and sexual function (Hubscher et al., 2018; Morrison et al., 2018). Previous work in animal models (Gad et al., 2014; Ward et al., 2014, 2016) and human SCI (Hubscher et al., 2018) indicate that sufficient excitation of the nervous system and/or residual supraspinal input, driven by repetitive stepping and appropriate sensory cues, resulted in improvements in multiple urological outcomes. Furthermore, the combination of locomotor training (step and stand training) plus spinal cord epidural stimulation (scES) has not only enhanced coordinated and controlled voluntary motor behavior, including walking over-ground in clinically motor complete SCI individuals (Grahn et al., 2017; Rejc et al., 2017; Angeli et al., 2018; Gill et al., 2018; Wagner et al., 2018; Darrow et al., 2019), but was also reported by individuals to improve physiologic outcomes such as cardiovascular function (Aslan et al., 2018; Harkema et al., 2018a,b; West et al., 2018) temperature regulation, bladder, and sexual function (Harkema et al., 2011; Darrow et al., 2019). Additional improvements in bladder storage and voiding have been reported with neuromodulation of the lumbosacral circuitry using scES following both motor complete (Herrity et al., 2018; Walter et al., 2018) and rodent models (Abud et al., 2015; Gad et al., 2016) of SCI. As the central state of excitability of the lumbosacral spinal cord is an important factor in promoting recovery of function (Angeli et al., 2014, 2018), the objective of this study was to test the effects of activity-dependent scES on bladder function in participants enrolled in scES training studies (activity-based recovery training, ABRT-scES) in our center relative to those participants in usual care who continued their typical daily lives without any study-related change in routine (no intervention). To further our understanding of the interaction between critical inter-dependent autonomic systems after SCI, the urological profiles in response to filling/emptying and cardiovascular-associated effects were also examined in a separate, large cross-sectional cohort.

## Materials and Methods

### Participants

A total of 85 individuals, 35 ± 11 years of age (71% male, 29% female), with chronic SCI are included in this study (Table 1). Study participant groups include a cross-sectional cohort (*n* = 65), a usual care cohort (*n* = 10), and an interventional cohort (*n* = 10). Participants in the cross-sectional cohort (*n* = 65) were enrolled in a research study (IRB#16.0179, NCT03364660, Task and Physiological Specific Stimulation for Recovery of Autonomic Function, Voluntary Movement and Standing using Epidural Stimulation and Training after Severe SCI) that was conducted between the years of 2017–2019 (Table 2). However, these individuals only participated in screening, including a urodynamic assessment, and did not continue into the next stage of the study which was usual care. Thus, cross-sectional and usual care cohorts are comprised of different individuals.

**Table 1:** Usual care and ABRT-scES participant characteristics.

Group	Participant	Age	Sex	Years post injury	Neuro level	AIS grade	Anal sensation	Bladder emptying method
Usual Care	A101	31	Male	2	C3	A	No	SP
	A100	51	Male	15	C3	A	No	SP
	A105	33	Male	9	C4	A	No	SP
	A109	41	Male	14	C4	A	No	CIC
	B38	20	Male	1	C4	B	Yes	CIC
	A123	28	Male	7	C4	B	Yes	CIC
	A119	24	Female	9	C5	A	No	CIC
	A110	21	Female	5	C5	A	No	SP
	B24	21	Male	2	C7	B	Yes	CIC
	B41	26	Male	8	C8	B	Yes	CIC
ABRT-scES	A68	35	Male	4	C2	A	No	CIC
	A80	33	Female	8	C3	A	No	SP
	A41	24	Male	7	C5	A	No	SP
	B21	31	Male	7	C5	B	Yes	CIC
	B23	33	Male	4	C6	B	Yes	SP
	B13	33	Male	4	C7	B	Yes	CIC
	B30	22	Female	3	T1	B	Yes	CIC
	A45	24	Male	2	T4	A	No	CIC
	A53	28	Male	2	T4	A	No	CIC
	A60	23	Male	3	T4	A	No	CIC

*ABRT, Activity-based recovery training; scES, spinal cord epidural stimulation; AIS, American Spinal Injury Association Impairment Scale; CIC, clean intermittent catheterization; SP, suprapubic catheter*.

**Table 2:** Cross-sectional participant characteristics.

		Bladder emptying method	
Number of participants	All *n* = 65	CIC *n* = 41	SP *n* = 24
Sex
Female	21 (32%)	8 (20%)	13 (54%)
Male	44 (68%)	33 (80%)	11 (46%)
Age (years)	37 ± 12	37 ± 12	36 ± 12
Years Post Injury	7 ± 6	7 ± 5	8 ± 6
Neuro Level Cervical	45 (69%)	23 (56%)	22 (92%)
Thoracic	20 (31%)	18 (44%)	2 (8%)
AIS Grade
A	38 (58%)	22 (54%)	16 (67%)
B	20 (31%)	14 (34%)	6 (25%)
C	5 (8%)	3 (7%)	2 (8%)
D	2 (3%)	2 (5%)	0 (0%)

*AIS, American Spinal Injury Association Impairment Scale; CIC, clean intermittent catheterization; SP, suprapubic catheter*.

Ten participants in the usual care cohort were enrolled in a research study conducted at the University of Louisville (IRB#16.0179, NCT03364660, *Task and Physiological Specific Stimulation for Recovery of Autonomic Function, Voluntary Movement and Standing using Epidural Stimulation and Training after Severe SCI*) between the years of 2017–2019. As part of that study, all participants received 2 Urodynamic assessments at least 5 months apart. This period was termed “usual care,” as the participants continued their typical daily lives without any study-related change in routine (no intervention). This phase addresses whether there would be any inherent variability between two Urodynamic measurements within the same time interval as the interventional cohort receiving scES and training.

Ten participants in the interventional cohort were enrolled for the current bladder study (IRB# 14.0062, NCT03036527 - R01) from other ongoing research studies at the University of Louisville investigating the effects of ABRT-scES on lower limb motor function (IRB #07.0066, NCT02339233, *Spinal Epidural Electrode Array to Facilitate Standing and Stepping in SCI*) and cardiovascular function (IRB #13.0625, NCT02037620, *Recovery of Cardiovascular Function with Epidural Stimulation after Human SCI*) between the years of 2010–2018. As part of the interventional studies, a 16-electrode array (5-6-5 Specify, Medtronic, Minneapolis, MN, USA) was surgically implanted at the T11-L1 vertebral levels over spinal cord segments L1-S1 as previously described (Harkema et al., 2011; Angeli et al., 2014). The electrode lead was tunneled subcutaneously and connected to the pulse generator (RestoreADVANCED, Medtronic, Minneapolis, MN, USA) placed ventrally in the abdomen. All research participants provided written, informed consent and the research was approved by the Institutional Review Board (University of Louisville, Louisville, KY, USA).

### Clinical Evaluation

All research participants received a clinical evaluation before study participation to assess motor and sensory status. Two clinicians independently performed the International Standards for Neurological Classification of SCI (Marino et al., 2003; Waring et al., 2010) to classify participants’ injuries using the ASIA (American Spinal Injury Association) Impairment Scale (AIS; Table 1). A physical examination also was performed by a clinician for medical clearance, ensuring participation safety using the following inclusion criteria: (1) stable medical condition; (2) no painful musculoskeletal dysfunction, unhealed fracture, contracture, pressure sore, or urinary tract infection that might interfere with training; (3) no untreated psychiatric disorders or ongoing drug abuse; (4) clear indications that the period of spinal shock is concluded determined by the presence of muscle tone, deep tendon reflexes or muscle spasms and discharged from standard inpatient rehabilitation; (5) non-progressive supra-sacral SCI; (6) bladder dysfunction as a result of SCI; and (7) epidural stimulator implanted at the lumbosacral spinal cord. None of the participants had ever received Botox injections for management of bladder dysfunction and all participants were off anti-spasticity medication (e.g., Baclofen). Note that all research participants refrained from taking any bladder medication at least 24 h before urodynamic testing [i.e., elimination half-life of oral oxybutynin, commonly used anticholinergic, is approximately 2 h and about 11 h to be eliminated from the body (Douchamps et al., 1988)] to rule out any pharmacologic impact on the clinical outcomes. Each participant also received a bladder/kidney Ultrasound at the time of enrollment and was medically cleared by both the study Urologist and study physician to participate in the research studies. None of the participants altered their method of bladder emptying throughout the study.

### Activity-Based Recovery Training

After implantation of the stimulator, 10 participants underwent a total of 160 sessions of activity-based recovery training (ABRT-scES). Six of those participants received alternating stand and step recovery-based training with scES. Stand training over-ground lasted 1 h per session (five sessions per week) and was always performed with spinal cord epidural stimulation using a custom-designed standing apparatus comprised of horizontal bars anterior and lateral to the individual to provide upper extremity assistance and balance support. The individual was encouraged to stand for as long as possible throughout the training session, with the goal of standing for 60 min with the least amount of assistance. Seated resting periods occurred when requested by the individual. If during standing, the participant’s knees or hips flexed beyond the normal standing posture, external assistance to facilitate hip and knee extension was provided either manually by a trainer or by elastic cords, which were attached between the two vertical bars of the standing frame. Step training (1 h, five sessions per week) was performed with bodyweight support (Innoventor, St. Louis, MO, USA) on a treadmill and always with spinal cord epidural stimulation. Research participants stepped at body-weight load and speed adapted to achieve appropriate stepping kinematics and trainers provided manual assistance only when needed following standard locomotor training principles (Harkema et al., 2012). Body-weight support was continuously reduced throughout the training sessions as the ability to bear weight on the weight-bearing limbs improved and manual facilitation was reduced as the ability to step independently improved. Four participants also underwent 160 sessions of cardiovascular training with scES which consisted of resting in a seated position for 2 h with continuous blood pressure and heart rate monitoring. Cardiovascular-scES configurations (anode and cathode electrode selection, voltage, frequency, and pulse width) were identified to maintain systolic blood pressure within a relatively stable blood pressure within non-injured defined normal ranges without eliciting motor activity (Harkema et al., 2018b). Participants in the cardiovascular-scES group also received 80 sessions of voluntary training with scES (included in the 160 sessions) which consisted of practicing, in the supine position, unilateral leg flexion, ankle dorsiflexion, and toe extension exercises with task-specific scES configurations daily (about 1 h per session, five sessions per week; Rejc et al., 2017). Note that use of scES at home during the 1-year before the follow-up assessment was variable and differed based upon sub-group (stand-scES only for locomotor training or cardiovascular-scES alone).

### Urodynamics

As described previously (Herrity et al., 2018; Hubscher et al., 2018), all data were obtained from standard urodynamic evaluations with recommendations from the International Continence Society (Blaivas et al., 1982; Schafer et al., 2002; Winters et al., 2012; Gammie et al., 2014). All assessments were conducted in the same manner, using the same equipment, and by a single research nurse. Using the Aquarius^®^ LT system (Laborie, Williston, VT, USA), cystometry was performed in the supine position *via* a single sensor, dual-channel catheter (7 Fr, T-DOC^®^ Air-Charged™, Laborie, Williston, VT, USA) with a continuous filling of sterile, body-temperature water (37°C) at a fixed slow rate of 20 ml/min. Abdominal pressure was measured *via* a rectal catheter (7 Fr, T-DOC^®^ Air-Charged™, Laborie, Williston, VT, USA). External anal sphincter electromyography (EMG; Neotrode II, Laborie, Williston, VT, USA) was recorded using surface patch EMG electrodes and a grounding pad was placed on a bony prominence, usually the hip or knee. Detrusor pressures were calculated by subtracting the intra-abdominal pressure from the intra-vesical pressure. Research participants were asked to cough to verify catheter positions and instructed to communicate sensations of a full bladder (first sensation), the desire to urinate (first urge to void), and strong desire to void, and the feeling that voiding/leaking cannot be delayed (maximum capacity; Wyndaele, 1998; Wyndaele and De Wachter, 2002). The volume of water and bladder pressure were recorded. Uninhibited bladder contractions also were identified. During the emptying phase, voluntary voiding events were generated from a low-pressure filling pressure and a proper detrusor contraction, distinguished from a reflexive leak, which often occurred in response to an elevation in detrusor pressure overriding the pressure generated at the bladder outlet. Participants were instructed to communicate bladder sensations (first sensation, desire, urgency) and any symptoms of autonomic dysreflexia (e.g., headache and/or chills). At the end of the filling, the was bladder drained with a catheter for measurement of residual volume. In the ABRT-scES group, filling cystometry was conducted post-implantation and before training, repeated after completion of 160 sessions of training, and at the 1-year follow-up time point. Stimulation was used only during daily ABRT and was not used during any of the cystometrogram evaluations.

Blood pressure and heart rate were obtained from the brachial artery, measured by the oscillometric technique (Carescape V100, GE Healthcare, Milwaukee, WI, USA), throughout the urodynamic session. As previously described (Aslan et al., 2018; Harkema et al., 2018b), noninvasive continuous blood pressure was also measured from a finger cuff by plethysmographic technique (ADInstruments). Brachial blood pressure was recorded at multiple time points during the study: (1) in the seated position when the participant presented to the lab; (2) supine position before catheter placement; (3) reclined position before filling with catheters in place; (4) continuously during testing; (5) post-filling to ensure blood pressure values returned to baseline; and (6) post-testing, once catheters were removed and the participant returned to his or her wheelchair. Any signs and self-reported symptoms of autonomic dysreflexia were documented and observed throughout testing. Bladder filling was ceased if any of the following conditions were observed: (1) spontaneous urine leakage; (2) filling ≥600 ml or reaching maximum bladder capacity as evidenced by a rise in the compliance curve; (3) high intravesical pressure ≥40 cmH_2_O or; (4) autonomic dysreflexia as evidenced by a sustained systolic blood pressure recording of ≥20 mm Hg from baseline and/or intolerable symptoms. If autonomic dysreflexia persisted beyond emptying, established guidelines were followed (Krassioukov et al., 2009). None of the research participants required the use of an antihypertensive agent to control autonomic dysreflexia after the assessment.

### Data Analysis

Bladder capacity was calculated as the volume of leaked or voided fluid plus any residual amount removed from the bladder. Note that the total volume also includes the excess amount produced through diuresis and not solely infused volume. Voiding efficiency (VE) was calculated as: VE = [volume voided/ (volume voided + residual volume) × 100]. Compliance was calculated by dividing the volume change (ΔV) by the change in detrusor pressure (ΔPdet) during that change in bladder volume and was expressed in ml/cm H_2_O (Abrams et al., 2002). Compliance is considered low below 20 cm/H_2_O (Stöhrer et al., 1999; Pannek et al., 2018). The intravesical pressure (Pves) at which involuntary expulsion of water/urine from the urethral meatus was observed was considered the detrusor leak point pressure (DLPP). Maximum detrusor pressure (MDP) was identified as the peak detrusor pressure during the voiding phase of the cystometrogram. Detrusor pressures were calculated by subtracting the intra-abdominal pressure from the intra-vesical pressure. All analyses were performed with customized software in MATLAB (MathWorks, Natick, MA, USA).

### Statistical Analysis

Continuous participant descriptors and bladder outcomes were tested for normality using the Kolmogorov–Simonov test. Variables that were found normally distributed were compared with two-sample *t*-test for two group comparisons or paired t-test for pre-post evaluations. Variables that failed the normality test compared with either the Rank Sum Test or the Signed Rank Test. Categorical variables were summarized with frequency count with associated percentage and compared with Chi-square tests or Fisher’s exact test as appropriate. Note that sample sizes were accounted for in all analyses, as 1 participant was not available for baseline Urodynamics and another participant was lost to follow-up. Both were in the ABRT-scES group. Blood pressure was also not available for 3 participants in the ABRT-scES group. All tests were 2-sided with a significance level of 0.05. Statistical analyses were performed in SAS 9.4 (SAS Inc., Cary, NC, USA).

## Results

### Clinical Characteristics

The clinical characteristics of the 20 research participants, all having motor complete SCI, are provided in Table 1. Features represented in the table were determined from the time at which each participant presented for either the post-implant/pre-training or pre-usual care Urodynamic assessment. The research participants’ mean age in the usual care and scES groups was 30 ± 10 and 29 ± 5 years, respectively, at the start of training. The usual care group included individuals having a cervical level of injuries, with a range of C3-C8 and a mean time since the injury of 7 ± 5 years. Individuals in the scES group had both cervical and upper thoracic level of injuries with a range of C2-T4 and a mean time since the injury of 4 ± 2 years. In the overall cohort, 80% of the participants were male, while 20% were female, closely representing the national statistical report of sex prevalence in SCI (National Spinal Cord Injury Statistical Center, 2020). Seven out of ten participants performed clean intermittent catheterization for bladder emptying, while the other three received a suprapubic cystostomy for bladder drainage. Note that suprapubic cystostomy in each of the three participants was performed at in-patient discharge and thus, each participant chronically utilized this method as the primary means of bladder emptying at study enrollment.

The clinical characteristics of the 65 participants in the cross-sectional cohort are provided in Table 2. The average age (37 ± 12 years) is more closely aligned with the national statistical report of the age at the time of injury (National Spinal Cord Injury Statistical Center, 2020). The mean time since injury (7 ± 6 years) was similar to those in usual care. About one-third of the population was female. The majority of individuals sustained a cervical level of injury and were motor and sensory complete, per AIS standards. The cohort was represented by 41 individuals performing intermittent catheterization and 24 with suprapubic cystostomy.

### Lower Urinary Tract Function—Storage Phase

**Figure 1:**
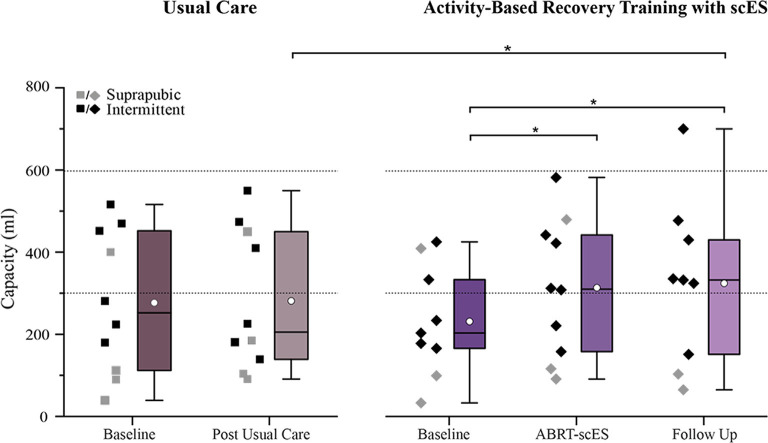
Impact of usual care (left) and activity-based recovery training (ABRT) with spinal cord epidural stimulation (scES; right) on bladder capacity. Bladder capacity significantly improved following ABRT-scES relative to baseline (*p* < 0.05). Capacity remained significant at the 1-year follow-up time point (*p* < 0.05) with a significant change from post-usual care (*p* < 0.01). Dashed horizontal lines represent the clinical reference range for bladder capacity (300–600 ml). Box plot represents the 25th percentile, the median, the 75th percentile, and the mean (white circle). ml, milliliters. *Indicates significance.

Baseline bladder capacity values were not statistically different between usual care and ABRT-scES groups (276 ± 174 ml and 231 ± 134 ml, respectively; Figure 1). Within the non-interventional usual care cohort alone, there were no significant changes in capacity at the post-usual care time point (281 ± 171 ml) relative to baseline. Following ABRT-scES, there was a significant improvement in bladder capacity relative to baseline (*p* < 0.05) that maintained significance at follow-up (*p* < 0.05; Baseline, 231 ± 134 ml; Post-training, 313 ± 166 ml; Follow-up, 324 ± 201 ml; Figure 1). Capacity values for 60% of the participants reached ranges within clinically recommended guidelines for bladder storage (range from 300–600 ml; Gray, 2001; Lukacz et al., 2011; Rosier et al., 2013) at both post-training and follow-up time points. There was also a significant improvement change in bladder capacity at post-training (70 ± 83 ml, *p* < 0.05) and at follow-up (102 ± 120 ml, *p* < 0.05) in the ABRT-scES group compared to post-usual care (5 ± 70 ml).

**Figure 2:**
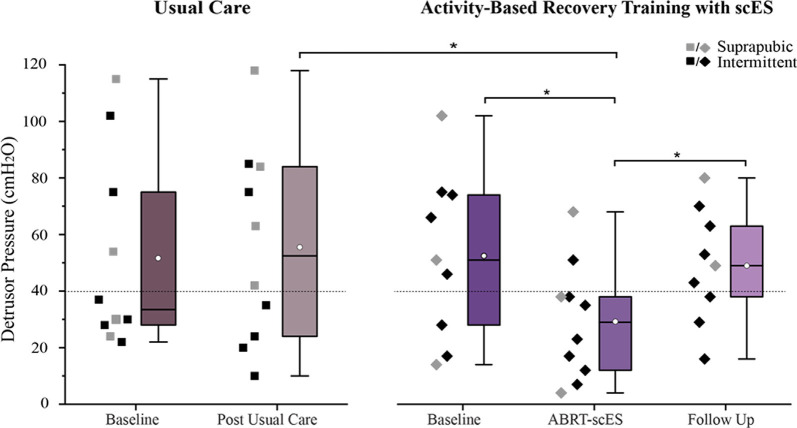
Reduction in detrusor pressure following activity-based recovery training with spinal cord epidural stimulation (ABRT-scES). The distribution of detrusor pressure values for each participant is presented for the usual care group (left) and the ABRT-scES group (right). Detrusor pressure was significantly improved (reduced) from baseline (*p* < 0.01) and the change in detrusor pressure was significantly improved relative to the post-usual care change (*p* < 0.05) following ABRT-scES. While detrusor pressure was significantly elevated at the 1-year follow-up time point (*p* < 0.01), it was unchanged from baseline values. There were no significant changes in detrusor pressure values in the usual care group. The dashed horizontal line (at 40 cmH_2_O) represents the critical cut-off value between high and low detrusor leak point pressure (DLPP). Box plot represents the 25th percentile, the median, the 75th percentile, and the mean (white circle). cmH_2_O, centimeters of water. *Indicates significance.

Baseline detrusor pressure values were not statistically different between usual care and ABRT-scES groups (53 ± 38 cmH_2_O and 53 ± 30 cmH_2_O, respectively; Figure 2). Within the non-interventional usual care cohort alone, there were no significant changes in detrusor pressure at the post-usual care time point (57 ± 38 cmH_2_O) relative to baseline. In contrast, detrusor pressure was significantly decreased post-training (Baseline, 53 ± 30 cmH_2_O; Post-training, 29 ± 20 cmH_2_O; *p* < 0.01), with the majority of participants (80%) having detrusor leak point pressure below 40 cmH_2_O (Figure 2). There was also a significant improvement change (reduction) in detrusor pressure at post-training (−22 ± 14 cmH_2_O, *p* < 0.006) in the ABRT-scES group compared to post-usual care (4 ± 21 cmH_2_O). However, at follow-up, detrusor pressure was significantly elevated relative to post-training values (49 ± 20 cmH_2_O, *p* < 0.01), and comparable to pre-training baseline (*p* > 0.05) in the ABRT-scES group.

**Figure 3:**
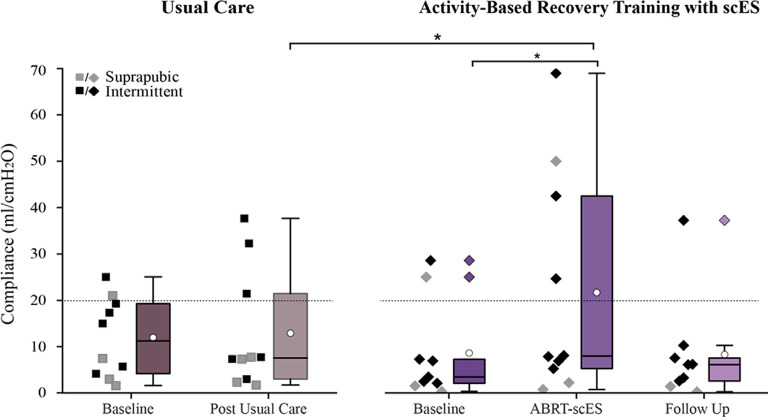
Comparison of bladder compliance values in participants receiving usual care (left) vs. activity-based recovery training with spinal cord epidural stimulation (ABRT-scES; right). Bladder compliance was significantly improved from baseline (*p* < 0.01) in the ABRT-scES group and the change in compliance was significantly improved relative to the post-usual care change (*p* < 0.05). Bladder compliance is compromised if it is below 20 ml/cmH_2_O (indicated by the dashed horizontal line). Box plot represents the 25th percentile, the median, the 75th percentile, and the mean (white circle). cmH_2_O, centimeters of water; ml, milliliters. *Indicates significance.

Similar to the post-training improvements in bladder capacity and pressure, bladder compliance significantly improved post-training (9 ± 11 ml/cmH_2_O vs. 20 ± 25 ml/cmH_2_O, *p* < 0.01; Figure 3), but reverted to baseline values by the follow-up (8 ± 11 ml/cmH_2_O). There were no changes in bladder compliance from baseline to post-usual care (12 ± 8 ml/cmH_2_O and 9 ± 11 ml/cmH_2_O).

### Lower Urinary Tract Function—Emptying Phase

The emptying phase of bladder function was assessed at the end of filling or when participants indicated a strong desire to void, typically reported as fullness in the lower abdominal region. In total, 4 participants (2 AIS A, 2 AIS B) receiving ABRT-scES demonstrated the ability to voluntarily void with intent during this study. One participant (AIS B) was able to partially empty her bladder at all three time points and thus, a uroflow was conducted before catheter placement and filling, and flow was captured during the voiding phase once the participant reached maximum capacity. At post-training, the maximum flow rate (Qmax) during emptying was 2.0 ml/s (12% voiding efficiency, VE). Note that the expected value for Qmax in females younger than 40 years of age is >22.0 ml/s (Walsh et al., 2002). Another participant (AIS B) partially voided post-training (11% VE) and at follow-up (36% VE). In two other participants (both AIS A), one voided post-training (9% VE), and the other at follow-up (17% VE). All four participants identified distinct sensations of bladder fullness (first sensation of filling, first desire, strong desire) guiding their report of the need to empty and their intent during the void attempt. In the ABRT-scES group, there were no significant changes in total voiding efficiency from pre-training (23 ± 27% VE) to post-training (28 ± 33% VE) nor from post-training to follow-up (24 ± 24% VE). None of the individuals in the usual care group were able to void voluntarily during testing. Voiding efficiency was also unchanged in the usual care group (10 ± 16% VE vs. 13 ± 29% VE).

### Blood Pressure Responses to Bladder Distention

**Figure 4:**
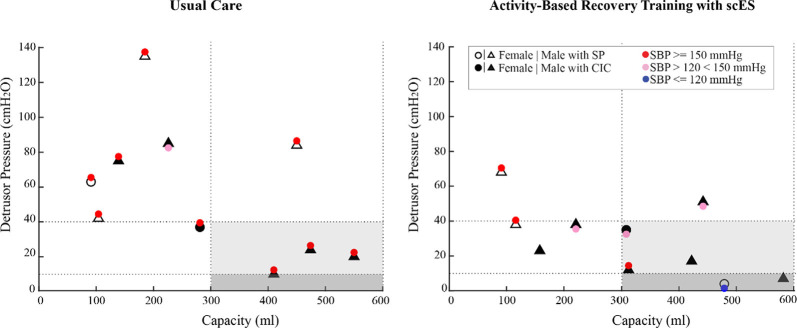
Bladder pressure-volume relationship and distention associated blood pressure responses in the usual care group (left) and the activity-based recovery training with spinal cord epidural stimulation (ABRT-scES) group (right) at post-usual care and post-training. While the ABRT-scES group experienced significantly lower blood pressure responses to bladder distention after training relative to usual care (*p* < 0.05), the majority of participants still responded with increased systolic blood pressure to bladder distention regardless of their bladder function (small or normal capacity and low or high detrusor pressure). The dashed horizontal line at 40 cmH_2_O represents the clinical upper threshold of high detrusor leak point pressure and the dashed horizontal line at 10 cmH_2_O represents the upper limit of detrusor filling pressure. The dashed vertical lines represent the clinical reference range for bladder capacity (300–600 ml). Blood pressure at maximum capacity was not available in three participants in the ABRT-scES group. CIC, clean intermittent catheterization; cmH_2_O, centimeters of water; ml, milliliters; mmHg, millimeters of mercury; SBP, systolic blood pressure; SP, suprapubic catheter.

In the interventional group, systolic blood pressure responses to bladder distention did not differ following ABRT-scES (Pre-training, 131 ± 15 mmHg; Post-training, 140 ± 13 mmHg), nor were there significant changes at follow-up (149 ± 26 mmHg) compared to baseline or post-training. Furthermore, the change in systolic blood pressure from pre-fill values (catheters in place) to values captured at the point of maximum cystometric capacity during the study indicates that ABRT-scES did not attenuate bladder-distention associated increases in systolic blood pressure (Pre-training change, 22 ± 20 mmHg; Post-training change, 25 ± 11 mmHg). However, concerning the usual care cohort, participants receiving ABRT-scES had significantly lower systolic blood pressure responses to bladder distention post-training (140 ± 13 mmHg, *p* < 0.05) compared to those in usual care (157 ± 18 mmHg; Figure 4). ABRT-scES sub-group (locomotor vs. cardiovascular + voluntary) training effects in relation to bladder outcomes were also evaluated. All pre-training bladder and blood pressure outcome measures between these two sub-groups were similar and there were no significant differences in these measures at pre-training, post-training, or follow-up.

### Cross-sectional Cohort—Urological Profiles in Chronic SCI

**Figure 5:**
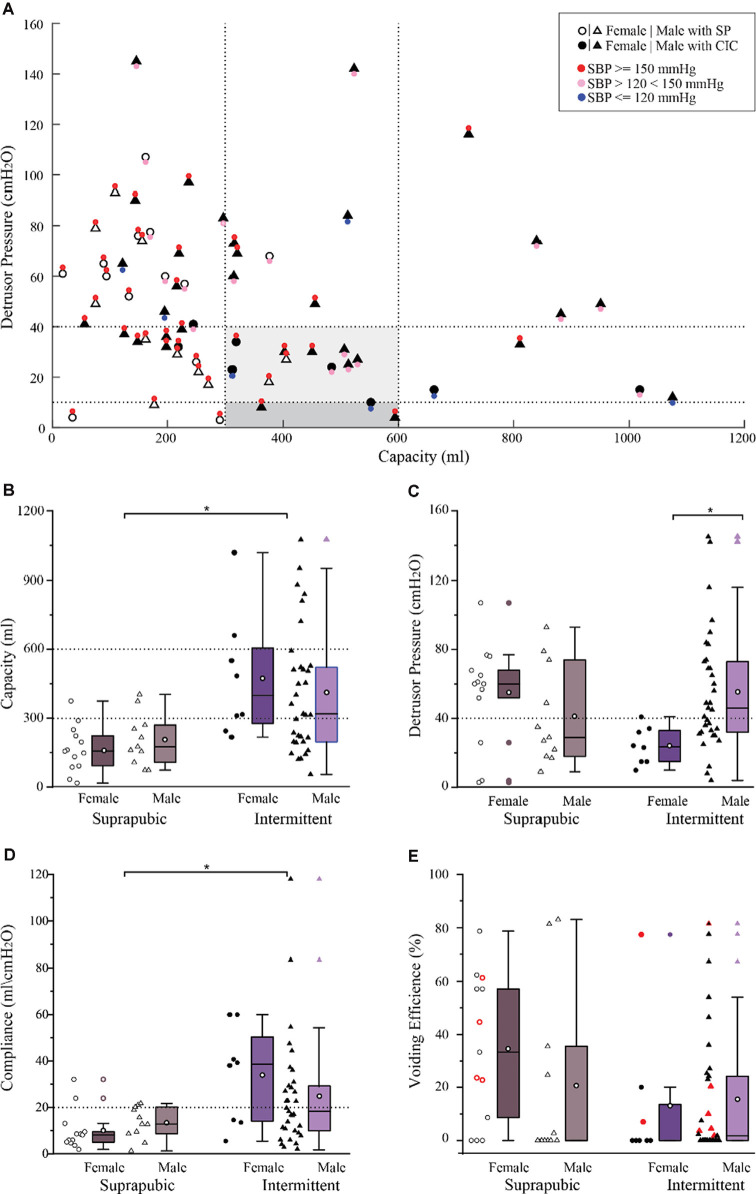
Bladder pressure-volume relationship with associated blood pressure responses and urodynamic outcomes in a large cross-sectional cohort (*n* = 65). **(A)** The bladder pressure-volume relationship and distention-associated cardiovascular interactions indicate that systolic blood pressure was above 150 mmHg (red circles) in 40 (62%) participants, less than 150 mmHg but higher than 120 mmHg (pink circles) in 18 (28%) participants, and within the normative range in only in seven (11%) participants (blue circles). **(B)** Bladder capacity overall was significantly lower in those with suprapubic catheters relative to those performing intermittent catheterization (*p* < 0.05). **(C)** Detrusor pressure was comparable between the suprapubic and intermittent groups showing that 34 (52%) participants had clinically high detrusor pressure associated with bladder filling. In those performing intermittent catheterization, detrusor pressure was significantly lower in female relative to male participants (*p* < 0.01). **(D)** Compliance was significantly lower in the suprapubic group compared to the intermittent catheterization group (*p* < 0.01). **(E)** Total voiding efficiency was variable between participants and includes both uncontrolled reflex voiding (leak) and voluntary voiding. Participants who were able to void voluntarily were indicated in red (*n* = 11) 17%. Box plot black horizontal lower, mid and upper lines represent the 25th percentile, the median, and the 75th percentile respectively. The white circle in the middle of the box represents the mean. CIC, clean intermittent catheterization; cmH_2_O, centimeters of water; ml, milliliters; mmHg, millimeters of mercury; SBP, systolic blood pressure; SP, suprapubic catheter.

Filling cystometry conducted on 65 research participants [*n* = 41 (63%), intermittent catheterization; *n* = 24 (37%) suprapubic catheter] revealed the majority of participants (*n* = 37, 57%) had low bladder capacity with values falling below normative ranges (300 ml; Gray, 2001; Lukacz et al., 2011; Rosier et al., 2013; Figure 5A). Within this sub-cohort (*n* = 23, 62%; 13 suprapubic catheters; 10 intermittent catheterizations), also had high detrusor pressure [>40 cmH_2_O; values above which are associated with upper tract deterioration (Rosier et al., 2013)]. The greatest blood pressure responses (>150 mmHg) were present in those using suprapubic catheters and having bladder capacity less than 300 ml [*n* = 17, (26%) of all participants]. The percentage of individuals from the cross-sectional cohort having the capacity and detrusor pressure within the recommended ranges (gray shaded region of Figure 5A) was only 20%, yet the vast majority (86%) still presented with elevated blood pressure responses (>120 mmHg). Conversely, a subset of participants (12%), all utilizing intermittent catheterization, had large bladder volumes above the upper limit of the normal capacity range (>600 ml).

In the overall cohort, those using intermittent catheterization had significantly greater capacity relative to those using suprapubic catheters (425 ± 266 ml vs. 186 ± 105 ml, respectively, *p* < 0.0001; Figure 5B). Yet, many using intermittent catheterization still had volumes below and above the recommended capacity values as well as elevated detrusor pressure. While there were no significant differences in detrusor pressure values between catheter groups (intermittent catheter, 49 ± 32 cmH_2_O; suprapubic catheter, 49 ± 29 cmH_2_O), both were higher than recommended safety values (Figure 5C). In the intermittent catheter group alone, despite the lower ratio of females (*n* = 8) to males (*n* = 33), females had significantly lower detrusor pressure than males (24 ± 11 cmH_2_O vs. 55 ± 35 cmH_2_O, *p* < 0.005; Figure 5C). No additional sex differences related to the urodynamic measures were identified in the overall cohort as well as within each catheter sub-type. Bladder compliance was significantly greater in the intermittent catheter group relative to the suprapubic group (27 ± 24 ml/cmH_2_O vs. 12 ± 8 ml/cmH_2_O, *p* = 0.002; Figure 5D). Total voiding efficiency for the entire cross-sectional cohort was low (20 ± 27%) and there were no differences between catheter groups (intermittent, 15 ± 24%; suprapubic, 28 ± 30%; Figure 5E). Blood pressure responses at maximum capacity were similar between those performing intermittent catheterization vs. those with suprapubic catheters (148 ± 25 mmHg vs. 159 ± 18 mmHg, respectively).

## Discussion

Regardless of bladder management type (indwelling vs. intermittent catheterization) the urologic presentation of individuals in the usual care cohort, the baseline profile of the ABRT-scES group, and the majority of participants in the cross-sectional cohort were similar and largely characterized by low bladder capacity, high detrusor pressure at maximum capacity, and ubiquitous high blood pressure elicited by bladder distention. Decreased bladder volumes and high pressures are associated with urinary over-activity, frequency, incontinence, and places one at risk for upper urinary tract deterioration. Furthermore, in susceptible individuals with uncontrolled sympathetic hyperactivity (injury above the mid-thoracic level), bladder distention is one of the primary triggers of autonomic dysreflexia. Episodes of autonomic dysreflexia have been reported to occur up to 40 times a day, dramatically increasing one’s risk for stroke by 300–400% (Cragg et al., 2013).

Despite the baseline presentation, we found that ABRT-scES positively influenced the storage phase, with improvements in bladder capacity, detrusor pressure, and overall compliance by the post-training time point. The increase in bladder capacity also persisted a year after training. Even though scES was not directly configured for bladder, nor was stimulation “on” during cystometry, sufficient excitation of neural networks, comprised of overlapping motor and autonomic networks, during training may have led to improved adaptations in detrusor activity and reciprocal somatic facilitation of the sphincter during bladder storage. While the ABRT-scES cohort demonstrated improved attenuation of blood pressure at maximum capacity relative to usual care, blood pressure was not entirely stabilized in response to bladder distention. We demonstrated that such elevations in blood pressure at maximum capacity were ubiquitous in a large cross-sectional cohort, indicating greater inter-connectedness of both the urinary and cardiovascular systems.

With the standard of care bladder management in the usual cohort, we did not see any change in bladder function across a similar time interval as those receiving the scES training intervention. While noted that all ten participants in the usual care cohort had a cervical level of injuries relative to the 60% with cervical injuries in the intervention group, there were no differences in baseline bladder outcome values across these two cohorts. Bladder management catheter usage was similar across the two groups (40% suprapubic—usual care; 30% suprapubic—ABRT-scES), and in fact, both cohorts had an equivalent percentage of participants with bladder capacity below 300 ml at baseline. In evaluating the impact of scES as an intervention, the ability to harness existing spinal neural control mechanisms with devices for bladder control has continued to evolve over time, as the lumbosacral circuitry controlling the bladder remains intact after most SCI’s. Some of the primary electrical stimulation approaches aimed at modulating bladder function have included stimulation of the spinal cord, select peripheral and sacral nerves, indirectly through the skin, as well as the bladder itself (McGee et al., 2015). Developed in the early 1980s, one of the more widely accepted treatments for refractory LUT dysfunction (i.e., urge urinary continence in those with bladder over-activity and non-obstructive urinary retention), chronic pelvic pain, as well as fecal incontinence, is sacral nerve stimulation with the Medtronic InterStim^®^ device (Tanagho and Schmidt, 1982; Schmidt, 1988). Within the SCI population, the utility of sacral nerve therapy has been moderately effective, primarily improving storage and emptying for those with chronic incomplete injuries (Lombardi and Del Popolo, 2009; Kessler et al., 2010). In complete SCI, however, implantation of bilateral sacral nerve stimulators acutely after injury was shown to prevent detrusor-over-activity and incontinence, suggesting early intervention may prevent irreversible effects attributed to LUT dysfunction (Sievert et al., 2010). While this approach appears to modulate micturition reflexes through peripheral afferent signaling mechanisms, based on the direct interaction between urinary and cardiovascular systems that is enhanced after SCI, neuromodulation of spinal cord networks with scES may provide an enhanced communication bridge between descending signals and residual circuits below the level of injury, thereby promoting coordinated autonomic responses that improve bladder function while stabilizing blood pressure. A sufficient central state of excitability may also be necessary so that afferent input and cues related to bladder filling as well as signals from supraspinal centers can drive efficient on-demand voiding.

In our center, the use of scES initially focused on modulating the excitability of spinal neural networks to enhance stepping, standing, and voluntary movement in response to provided task-specific sensory cues in both complete and incomplete SCI (Harkema et al., 2011; Angeli et al., 2014). The integration of somatosensory and residual descending inputs to the spinal circuitry further contributed to unexpected gains in other physiological systems such as the bladder, sexual function, and temperature regulation (Harkema et al., 2011). Even though stimulation parameters were aimed at influencing the motor system and the execution of specific motor patterns, multiple autonomic improvements occurred. With the activation of lumbosacral spinal networks through ABRT-scES, significant improvements in bladder capacity, and detrusor pressure (reduction) were achieved. The finding that capacity remained significantly increased from baseline at follow-up may likely be due to participant clearance for community integration and independent home-training after completion of the intervention phase, whereby they utilize scES for standing or cardiovascular function consistently, and thus continue to activate these overlapping circuits. Other factors related to urological care that cannot be controlled outside the research environment, such as the method of bladder emptying (indwelling vs. intermittent catheterization) or medication usage impacting detrusor contractility, may be associated with the long-term changes in detrusor pressure at follow-up. Importantly, however, detrusor pressure never worsened (increased) in any of the research participants in response to scES, which has previously been suggested (Beck et al., 2020). Our results support the effect of adaptive scES training-induced plasticity in the nervous system and the ability of the spinal cord to interpret and integrate distinct somatosensory cues associated with loading and/or autonomic inputs (Wolpaw and Tennissen, 2001). A vesico-somatic interaction between the circuitries controlling bladder and locomotor function is also anticipated, as we have previously demonstrated that locomotor training alone was sufficient to induce significant improvements in multiple bladder parameters (Hubscher et al., 2018). In the ABRT-scES group, there were improvements in blood pressure responses elicited from bladder distention relative to the usual care cohort, albeit, still not within normative ranges (i.e., 110–120 mmHg). It is important to note that a great deal of hemodynamic instability occurs after SCI, independent of level and severity of injury, and thus, understanding variations in blood pressure and heart rate responses among individuals with SCI will improve the characterization and overall clinical care of autonomic dysfunction (Wang et al., 2020). Potential factors contributing to the scES training-induced reduction of autonomic dysreflexia associated with bladder distention may be indirectly linked with central suppression of C-fiber mediated bladder reflex activity (de Groat, 1995) as the detrusor smooth muscle becomes more compliant in response to mechanical stimuli and scES with long-term training. In a rodent model of SCI, such C-fiber bladder afferents (capsaicin-sensitive) have been implicated in the generation of detrusor overactivity and non-voiding contractions (primary triggers of autonomic dysreflexia) during the filling phase (Cheng et al., 1995).

The study of a large cohort of individuals with chronic SCI revealed that the vast majority of participants had an altered relationship between bladder volume and bladder pressure. Similar to the urological profiles in the usual care group and baseline ABRT-scES values, these participants also demonstrated storage capacity and detrusor pressure abnormalities, ranging from very small volumes to large distended bladders, as well as ubiquitous high blood pressures regardless of bladder management type. Critical to ensuring the long-term safety of the upper and LUT is the ability to achieve and maintain safe storage pressures. The use of indwelling suprapubic catheters as a method to continuously drain the bladder is an alternative method of emptying the bladder if self-intermittent catheterization poses a challenge. While suprapubic catheters are regarded by many consumers as a convenient, effortless alternative to a more demanding urethral catheterization management protocol (Ahluwalia et al., 2006), constant bladder drainage through an open tube to an external storage bag impairs the physiological cyclic pattern of storage and emptying, resulting in possible histological changes and poor functional compliance (Pannek et al., 2010). As a result, minor increases in bladder volume may illicit high blood pressure. As expected, there was greater cardiovascular responsiveness to bladder distention during cystometry in this population. The rapid increase in systolic blood pressure is likely more dramatic in those utilizing suprapubic catheters, as these individuals represent a majority having cervical or high thoracic SCI, resulting in the loss of supraspinal regulation of spinal sympathetic activity and disrupted cardiovascular regulation (Garstang and Miller-Smith, 2007). Bladder distention is one of the primary triggers of autonomic dysreflexia and such severe fluctuations in blood pressure pose a major limitation in the ability to recover bladder function long-term and place these individuals at cardiovascular risk. Overall bladder self-care and hygiene, including routine suprapubic catheter replacement, caregiver availability to assist with catheter maintenance, and the incidence of LUT comorbidities (e.g., urinary tract infections) may also be contributing factors in diverse urological outcomes evident in those using suprapubic catheters.

A select group of individuals was also found to have over-distended bladders, with high bladder volumes, characterized as areflexic (low detrusor tone). Oftentimes, a reduction in the standard frequency of daily and/or nightly catheterizations as a means to curtail emptying can contribute to bladder over-distention long-term (Consortium for Spinal Cord, Medicine, 2006). One such contributing factor is altered diurnal secretion of antidiuretic hormone after SCI (Szollar et al., 1997; Kilinc et al., 1999), resulting in the incidence of polyuria (overproduction and/or passage of urine). Excessive urine production can further exacerbate an already demanding catheterization schedule and disrupt daily life. We have found that the mechanisms underlying SCI-induced polyuria are multifactorial, including an interplay of various peptides involved in the physiological regulation of fluid balance, plasma volume, and overall urine output (Ward and Hubscher, 2012; Montgomery and Hubscher, 2018; Gumbel et al., 2020).

Given that the consequences of SCI affect multiple systems, scES as an intervention has the potential to benefit autonomic systems and organ function, dramatically impacting quality of life. Further gains in bladder control may be achieved by accessing these highly integrated networks using scES parameters directly targeted for optimizing storage and emptying while regulating cardiovascular function, as bladder distention is a major trigger for autonomic dysreflexia. A multi-pronged neurorehabilitative approach that builds upon the principles of task-specific training provides an avenue to facilitate the recruitment of both spinal circuitries and spared supraspinal connections important for recovering function after chronic SCI (Rejc and Angeli, 2019). Further research on the mechanisms driving the effects of scES on bladder function will significantly advance the technology and therapeutic approaches for bladder management, dramatically improving the quality of life for people with SCI.

## Data Availability Statement

The raw data supporting the conclusions of this article will be made available by the authors, without undue reservation.

## Ethics Statement

The studies involving human participants were reviewed and approved by The University of Louisville Institutional Review Board. The participants provided their written informed consent to participate in this study.

## Author Contributions

AH, CH, and SA contributed to the acquisition of data. AH, SA, and BU contributed to the analysis of data. AH drafted the manuscript. SA developed programming tools, including acquisition software and coding for cystometry analyses and data visualization. BU conducted the statistical analyses. AM contributed to medical oversight and provided the clinical interpretation of data. AH, SA, CH, and SH contributed to concept development, design, and data interpretation. CH and SH obtained funding and supervised the research. All authors critically reviewed and revised the manuscript. All authors contributed to the article and approved the submitted version.

## Conflict of Interest

The authors declare that the research was conducted in the absence of any commercial or financial relationships that could be construed as a potential conflict of interest.
